# An endangered wild tea plant resource with unique alkaloid and catechin profiles and potential high-quality tea

**DOI:** 10.1093/hr/uhag031

**Published:** 2026-01-30

**Authors:** Dingding Liu, Yuanquan Chen, Jiedan Chen, Chenyu Zhang, Yuanyuan Ye, Piao Mei, Peixin Wang, Shiqi Ding, Yang Gong, Junyu Wang, Xinrong Hu, Mingzhe Yao, Chunlei Ma

**Affiliations:** State Key Laboratory of Tea Plant Germplasm Innovation and Resource Utilization, Tea Research Institute of the Chinese Academy of Agricultural Sciences, Hangzhou 310008, China; Guangxi South Subtropical Agricultural Sciences Research Institute, Chongzuo 532415, China; State Key Laboratory of Tea Plant Germplasm Innovation and Resource Utilization, Tea Research Institute of the Chinese Academy of Agricultural Sciences, Hangzhou 310008, China; State Key Laboratory of Tea Plant Germplasm Innovation and Resource Utilization, Tea Research Institute of the Chinese Academy of Agricultural Sciences, Hangzhou 310008, China; State Key Laboratory of Tea Plant Germplasm Innovation and Resource Utilization, Tea Research Institute of the Chinese Academy of Agricultural Sciences, Hangzhou 310008, China; State Key Laboratory of Tea Plant Germplasm Innovation and Resource Utilization, Tea Research Institute of the Chinese Academy of Agricultural Sciences, Hangzhou 310008, China; State Key Laboratory of Tea Plant Germplasm Innovation and Resource Utilization, Tea Research Institute of the Chinese Academy of Agricultural Sciences, Hangzhou 310008, China; State Key Laboratory of Tea Plant Germplasm Innovation and Resource Utilization, Tea Research Institute of the Chinese Academy of Agricultural Sciences, Hangzhou 310008, China; State Key Laboratory of Tea Plant Germplasm Innovation and Resource Utilization, Tea Research Institute of the Chinese Academy of Agricultural Sciences, Hangzhou 310008, China; State Key Laboratory of Tea Plant Germplasm Innovation and Resource Utilization, Tea Research Institute of the Chinese Academy of Agricultural Sciences, Hangzhou 310008, China; Jinhua Academy of Agricultural Sciences, Jinhua 321000, China; State Key Laboratory of Tea Plant Germplasm Innovation and Resource Utilization, Tea Research Institute of the Chinese Academy of Agricultural Sciences, Hangzhou 310008, China; State Key Laboratory of Tea Plant Germplasm Innovation and Resource Utilization, Tea Research Institute of the Chinese Academy of Agricultural Sciences, Hangzhou 310008, China

Dear Editor,

Wild tea plants are a kind of precious germplasm resource in China. However, their survival is increasingly threatened by a lack of effective management and conservation strategies. *Camellia fangchengensis* (FCC), a wild tea species endemic to Guangxi, discovered in 1981, was formally named by Liang Shengye and colleagues. Due to its extremely limited distribution and small population, FCC has been listed in the first batch of the National Key Protected Wild Plants Directory [1]. Since its discovery, research on this species has remained scarce. In 2023, our team revisited the area near Huashi Town, Fangchenggang, Guangxi, and relocated this rare resource. Over four decades, ecological changes, human interference, and declines in natural populations have dramatically reduced its numbers, and now only a few dozen individuals remain. To better understand and utilize this resource, we performed a comprehensive investigation of its morphology, biochemical composition, key metabolic mechanisms, and processing suitability for tea production. Our findings provide novel insights into tea genetics and processing, and highlight the untapped potential of wild tea species for improving tea quality and diversifying product lines.

We systematically described the morphological characteristics of *C. fangchengensis* for the first time. It is a small arbor with extra-large leaves, on which the young branches, abaxial leaf surfaces, and calyx are densely covered with trichomes (Fig. 1A). Microscopic observations revealed a single layer of cells in both the upper and lower epidermis, a thick cuticle, and a single layer of compact palisade tissue cells (Fig. S1). These features differed from other tea plants in the genus *Camellia*, Section *Thea*. We quantified major biochemical components in tender shoots from four FCC individuals (FCC_1 to FCC_4) and four *C. sinensis* cultivars (BY1, JS2, ZJ, LJ43) (Fig. 1B). FCC displayed a notably high catechin C (C) content (5.68%), followed by ECG, while its EGCG content was extremely low (<1%). FCC also exhibited extremely high theobromine (TB, 4.08%–5.34%) and very low caffeine (CAF, 0.25%–0.45%) levels compared with *C. sinensis*. Furthermore, FCC contained a high proportion of dihydroxy–catechins and a low proportion of trihydroxy–catechins, a pattern in contrast that of *C. sinensis* (Fig. 1C). These results demonstrate that FCC possesses a unique catechin profile, even among wild tea resources.

To further investigate the underlying metabolic mechanisms, we performed metabolomic and transcriptomic analyses using new shoots (NS, NF) and mature leaves (MS, MF) from both *C. sinensis* (LJ43) and *C. fangchengensis* (FCC_1). Principal component analysis of the metabolome and Pearson’s correlations of transcriptome data revealed strong clustering patterns within each group and high correlation among biological replicates (Fig. 1D and G). A total of 487 common differentially accumulated metabolites (DAMs) were identified from metabolomic data between NS vs. NF group and MS vs. MF group. Meanwhile, we also screened 5312 common differentially expressed genes (DEGs) in these two comparison groups based on transcriptomic data (Fig. 1E, F, and  H; Table S1). KEGG enrichment analysis revealed that the majority of shared DAMs and DEGs were significantly enriched in ‘metabolic pathway’. Besides, some common DEGs were mapped to ‘biosynthesis of amino acids’ and ‘biosynthesis of secondary metabolites’. And some common DAMs were significantly enriched in the ‘biosynthesis of phenylpropanoids’, ‘flavonoid biosynthesis’, and ‘caffeine metabolism’.

**Figure 1:**
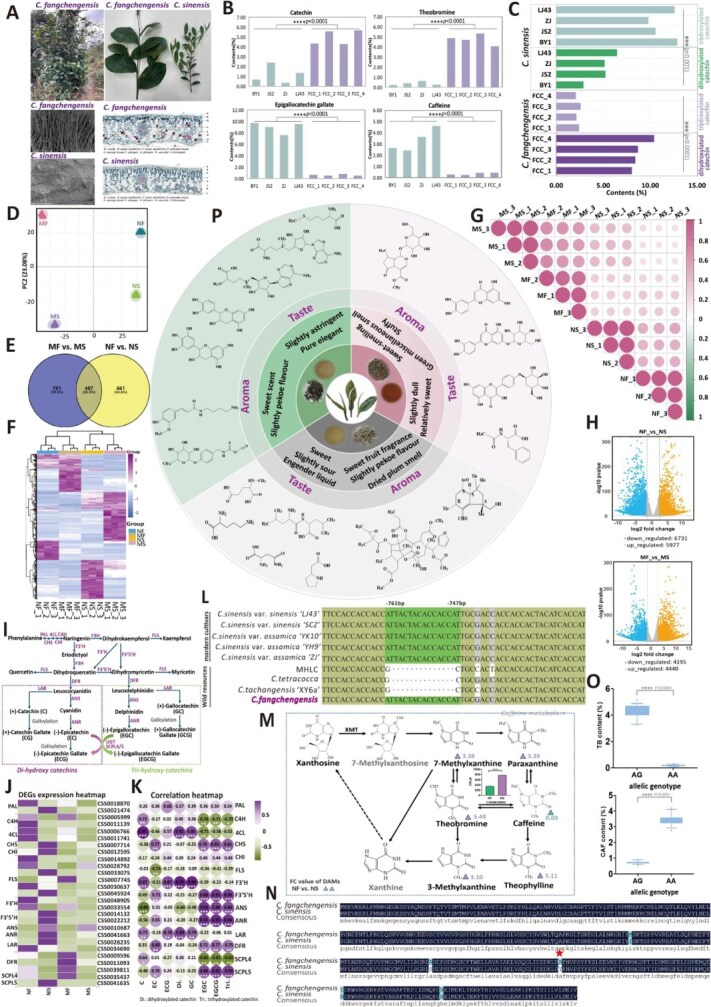
Comprehensive analyses of *Camellia fangchengensis*. **A**. Morphological characteristics of *C. fangchengensis*. (phenotypic traits; scanning electron microscopy (SEM) images of leaves from *C. fangchengensis* and *C. sinensis*; tissue section images of leaves from *C. fangchengensis* and *C. sinensis*). **B.** Biochemical composition analysis of *C. fangchengensis* and *C. sinensis*. **C.** Comparative analysis of di- vs. tri-hydroxylated catechin contents in *C. fangchengensis* and *C. sinensis*. **D.** Principal component analysis of metabolic profiles in mature leaves (MF, MS) and new shoots (NF, NS) from *C. fangchengensis* and *C. sinensis*. **E.** Venn diagram of DAMs between NF vs. NS and MF vs. MS. **F.** Heatmap of common DAMs identified in NF vs. NS and MF vs. MS. **G.** Pearson’s correlation analysis of transcriptome data from mature leaves (MF, MS) and new shoots (NF, NS) in *C. fangchengensis* and *C. sinensis*. **H.** Volcano plots of differentially expressed genes (DEGs) in NF vs. NS and MF vs. MS. **I.** Catechin metabolic pathway in tea plants. **J.** Heatmap of expression patterns for 27 DEGs involved in catechin biosynthesis in mature leaves (MF, MS) and new shoots (NF, NS) from *C. fangchengensis* and *C. sinensis*. **K.** Correlation heatmap between DEGs and catechin components. **L.** A 14 bp indel variation of *F3′5′H* promoters in wild and cultivated tea plants. **M**. Differential accumulation of metabolites and expression patterns of the *CsTCS1* gene. **N**. Sequence comparison of the *CsTCS1* gene between *C. fangchengensis* and *C. sinensis*. **O.** Caffeine and theobromine contents in tea plants with two different *CsSNP36772011* genotypes. **P.** Sensory evaluation and metabolic profiling of green, white, and black teas produced from *C. fangchengensis* new shoots.

Trihydroxy catechins are typically more abundant than dihydroxy catechins in most Chinese tea germplasm [2], whereas FCC shows the opposite pattern, suggesting the presence of a distinct regulatory mechanism. Transcriptome analysis identified 27 DEGs encoding 13 enzymes involved in flavonoid metabolism (Fig. 1I and J, Table S2, and Table S3). Pearson’s correlation analysis between these DEGs and eight catechin components revealed strong associations: EGCG and trihydroxylated catechins correlated positively with *F3′5′H*, *ANR*, and *SCPL5* (*r* = 0.90–0.95), whereas catechin C and dihydroxylated catechins correlated with *4CL*, *F3′H*, and *LAR* (*r* = 0.87–0.99) (Fig. 1K). Notably, *F3′H* (*CSS0048905*) expression in FCC (NF_FKPM: 137.61) was ~5-fold higher than in *C. sinensis* (NS_FKPM: 27.75), likely contributing to its elevated dihydroxy–catechin levels (Fig. 1J; Table S3). Conversely, *F3′5′H* (*CSS0022212*) showed high expression in *C. sinensis* (NS_FKPM: 748.09) but low expression in FCC (NF_FKPM: 9.31) (Fig. 1J; Table S3). These findings indicate that *F3′H* and *F3′5′H* play key roles in directing metabolic flux toward dihydroxy- versus trihydroxy–catechin biosynthesis. To enable more accurate sequence comparison, the Illumina sequencing data of FCC were *de novo* assembled using Jellyfish software. The preliminary assembly was approximately 2.99 Gb in size, with an estimated heterozygosity of 1.60% (Table S4). Synteny analysis between the chromosome-level ‘Shuchazao’ genome and the *de novo* assembled FCC genome revealed strong collinearity and allowed us to identify the FCC homologs of *F3′5′H* (*Cfa11G024720*) and *F3′H* (*Cfa15G017540*) (Fig. S3). Sequence alignment and phylogenetic analysis showed that *CSS0022212* shared 99.80% amino acid identity with *Cfa11G024720*, and *CSS0048905* shared 99.03% identity with *Cfa15G017540* (Fig. S4). We then cloned and Sanger-sequenced *F3′5′H* and *F3′H* from FCC and *C. sinensis*, confirming high-sequence similarity (Fig. S5). Notably, unlike other wild tea species, the promoter region of *F3′5′H* in FCC does not contain a previously reported 14-bp deletion (Fig. 1L), suggesting that the reduced expression of *F3′5′H* in FCC may be regulated by a distinct mechanism [3].

Caffeine is the predominant alkaloid in most tea cultivars, typically ranging 2%–5%. Although secondary metabolites in tea offer health benefits, the stimulating effect of caffeine can cause insomnia in caffeine-sensitive individuals. Caffeine synthase 1 (*TCS1*) is the key enzyme that converts theobromine to caffeine [4, 5]. We hypothesized that sequence variations in the *TCS1* gene might reduce or eliminate this enzymatic activity in FCC. Transcriptome analysis of new shoots revealed expression levels of the *TCS1* gene (*CSS0032602*) to be twice as high in *C. sinensis* as in FCC (Fig. 1M). Sequence alignment identified 12 SNPs, five of which were missense mutations (Fig. 1N). We genotyped 40 FCC and 40 *C. sinensis* individuals (from hybrid population ‘Longjing 43’ × ‘Huangjinya’) using Kompetitive allele-specific PCR assays, and discovered a novel SNP (*CsSNP36772011*) with an AG genotype in FCC and an AA genotype in 1*C. sinensis* (Fig. 1O, Tables S5, and  S6). This SNP caused a threonine-to-alanine substitution and was significantly associated with increased theobromine and reduced caffeine content.

To explore the practical applications of FCC, we processed its new shoots into green, black, and white teas using traditional methods. Green tea exhibited a sweet aroma, mild pekoe flavor, and slight astringency. Black tea had a sweet aroma and a mellow but slightly dull flavor. Remarkably, white tea featured a distinctive sweet fruit fragrance with a dried plum note, slight pekoe flavor, and a sweet, lingering aftertaste. Among the three, white tea showed the highest quality characteristics (Fig. 1P; Table S7). UPLC-MS/MS analysis identified 2381 nonvolatile metabolites across 15 classes, revealing distinct metabolite profiles in the three tea types (Table S8). White tea showed significantly higher levels of amino acids and derivatives (e.g., homoproline, L-asparagine, L-ornithine, N-methyl-L-glutamate) that contribute to its sweetness. Flavonoids and alkaloids—typically responsible for bitterness—were present in much lower amounts in white tea. Terpenoids, key aroma compounds, were also more abundant in white tea, including compounds such as (3R,4R)-p-Menth-1-ene-3,4-diol-3-O-β-D-glucoside and Swietenialide D, explaining its unique fruity and dried plum aroma.

In summary, we comprehensively characterized *C. fangchengensis* for the first time. Our findings demonstrate that FCC has a distinct biochemical profile, with high theobromine and low caffeine levels, and is dominated by catechin (C) rather than EGCG. The expression of key flavonoid biosynthetic genes, particularly *F3′H* and *F3′5′H*, likely contributes to its unique catechin profile. A novel missense mutation (*CsSNP36772011*) in the *TCS1* gene appears to underlie its low caffeine and high theobromine content. Moreover, FCC white tea exhibits excellent sensory qualities, making it a strong candidate for high-end tea production. This study both expands our understanding of FCC’s morphology and metabolism, and underscores the practical potential of wild tea resources for commercial development.

## Supplementary Material

Web_Material_uhag031

## Data Availability

The transcriptome data of FCC and LJ43 can be found in the NCBI Sequence Read Archive (SRA) under the bioproject number PRJNA1308736. The illumina sequencing data of FCC can also be found in the NCBI SRA under the bioproject number PRJNA1395793.The other relevant data can be found within the manuscript and its supplementary information. Supplementary Data is available at http://www.teaplant.top/teagvd/download.
