# Supplementary material for: An endangered wild tea plant resource with unique alkaloid and catechin profiles and potential high-quality tea
Source: Hortic Res. 2026 Jan 30;13(5):uhag031. doi: 10.1093/hr/uhag031 (PMC13148153; doi:10.1093/hr/uhag031)
Supplement: Web_Material_uhag031 [file web_material_uhag031.docx]

***Supplementary Data 1***

An endangered wild tea plant resource with unique alkaloid and catechin profiles and potential for high-quality tea

Dingding Liu^1†^, Yuanquan Chen^2†^, Jiedan Chen^1^, Chenyu Zhang^1^, Yuanyuan Ye^1^, Piao Mei^1^, Peixin Wang^1^, Shiqi Ding^1^, Yang Gong^1^, Junyu Wang^1^, Xinrong Hu^3^, Mingzhe Yao^1^*, Chunlei Ma^1^*

^1^ State Key Laboratory of Tea Plant Germplasm Innovation and Resource Utilization, Tea Research Institute of the Chinese Academy of Agricultural Sciences, Hangzhou 310008, China.

^2^ Guangxi South Subtropical Agricultural Research Institute, Chongzuo 532415, China

^3^Jinhua Academy of Agricultural Sciences, Jinhua 321000, China

*Corresponding authors. E-mail: malei220@mail.tricaas.com; [yaomz@tricaas.com](mailto:yaomz@tricaas.com)

†These authors contributed equally to this work.

**Supplementary Table**

| **Sample** | **Raw Reads** | **Clean Reads** | **Reads**  **mapped** | **Unique mapped** | **Error Rate(%)** | **Q20(%)** | **Q30(%)** | **GC Content(%)** |
| --- | --- | --- | --- | --- | --- | --- | --- | --- |
| NF_1 | 47370128 | 46117950 | 37831044(82.03%) | 31880181(69.13%) | 0.02 | 98.11 | 94.54 | 45.02 |
| NF_2 | 47828344 | 46482930 | 37716441(81.14%) | 31693450(68.18%) | 0.03 | 97.82 | 93.9 | 45.33 |
| NF_3 | 50719210 | 48679844 | 38798625(79.70%) | 32510291(66.78%) | 0.03 | 97.49 | 93.33 | 45.53 |
| NS_1 | 46295280 | 45307272 | 40339239(89.03%) | 33794471(74.59%) | 0.02 | 98.08 | 94.43 | 45.27 |
| NS_2 | 45391714 | 44337758 | 39377101(88.81%) | 33027037(74.49%) | 0.02 | 98.07 | 94.39 | 45.25 |
| NS_3 | 46978748 | 45973646 | 40834857(88.82%) | 34375896(74.77%) | 0.03 | 97.88 | 93.97 | 45.25 |
| MF_1 | 44893364 | 42539366 | 35314608(83.02%) | 29397760(69.11%) | 0.03 | 96.62 | 91.15 | 44.71 |
| MF_2 | 51369366 | 48023182 | 39354533(81.95%) | 32768172(68.23%) | 0.03 | 97.16 | 92.42 | 45.57 |
| MF_3 | 52234924 | 49798244 | 40440384(81.21%) | 33789458(67.85%) | 0.03 | 96.98 | 92.07 | 44.06 |
| MS_1 | 50338316 | 47963636 | 43107862(89.88%) | 35949494(74.95%) | 0.03 | 97.39 | 92.85 | 44.85 |
| MS_2 | 44004410 | 41981116 | 37560681(89.47%) | 31399291(74.79%) | 0.03 | 97.11 | 92.22 | 44.6 |
| MS_3 | 52328970 | 48310952 | 42850327(88.70%) | 35975855(74.47%) | 0.04 | 95.48 | 88.1 | 44.12 |

Table S2 Statistical information of the transcriptome data of new shoots and mature leaves of FCC and LJ43

Table S3 Common DEGs involved in catechin biosynthesis of new shoots and mature leaves of FCC and LJ43

| ID | NF_1 | NF_2 | NF_3 | NS_1 | NS_2 | NS_3 | MF_1 | MF_2 | MF_3 | MS_1 | MS_2 | MS_3 |
| --- | --- | --- | --- | --- | --- | --- | --- | --- | --- | --- | --- | --- |
| CSS0018870 | 26.3367 | 28.8641 | 22.8121 | 5.4503 | 6.5582 | 8.5138 | 14.9652 | 12.8589 | 10.3134 | 1.9475 | 1.6697 | 1.8209 |
| CSS0021474 | 38.2298 | 47.6091 | 48.36 | 298.5519 | 280.6774 | 293.2759 | 18.614 | 22.4571 | 22.2849 | 62.7647 | 64.9726 | 53.7327 |
| CSS0005999 | 35.6223 | 20.0796 | 19.6184 | 1.2946 | 1.6327 | 2.2765 | 47.3856 | 20.7275 | 17.1852 | 0.9663 | 1.5849 | 2.2795 |
| CSS0011139 | 24.3228 | 22.7934 | 22.9757 | 0.0796 | 0.2438 | 0.039 | 1.0977 | 0.8066 | 1.2863 | 0 | 0.0448 | 0 |
| CSS0006766 | 45.6892 | 47.535 | 52.9549 | 11.2145 | 12.7485 | 12.2715 | 22.2153 | 28.8019 | 29.3528 | 7.4667 | 9.1515 | 10.1711 |
| CSS0011741 | 32.9928 | 34.1762 | 31.9226 | 4.4388 | 5.4826 | 5.598 | 18.2356 | 12.3077 | 10.4989 | 0.8484 | 1.4841 | 1.5641 |
| CSS0007714 | 77.9859 | 76.9506 | 67.7477 | 379.6948 | 385.255 | 394.3273 | 58.8834 | 32.0923 | 23.231 | 120.3546 | 124.6876 | 103.5937 |
| CSS0012595 | 16.596 | 19.562 | 23.8824 | 47.6731 | 47.6417 | 45.2413 | 3.5723 | 4.5911 | 5.2223 | 14.2956 | 13.2375 | 17.0823 |
| CSS0014892 | 24.8273 | 25.6534 | 34.6646 | 0.5873 | 1.2491 | 1.9187 | 1.7309 | 1.8493 | 1.6342 | 0.0717 | 0.0551 | 0.0977 |
| CSS0028792 | 31.665 | 24.9193 | 25.5177 | 9.7005 | 10.6114 | 7.1309 | 5.1575 | 1.8973 | 3.0477 | 18.656 | 19.8448 | 19.5889 |
| CSS0033075 | 16.8208 | 15.6041 | 14.5576 | 402.6933 | 400.0254 | 418.4205 | 13.7962 | 12.1496 | 9.408 | 46.5986 | 47.8159 | 31.0058 |
| CSS0007745 | 4.6495 | 3.5477 | 3.3763 | 47.9912 | 48.4328 | 52.018 | 175.1488 | 123.4518 | 117.8043 | 34.2746 | 32.0033 | 32.1749 |
| CSS0030637 | 34.7033 | 30.9766 | 26.9781 | 11.805 | 12.3382 | 8.6093 | 1.8111 | 1.1643 | 1.9888 | 4.1232 | 3.8079 | 4.2148 |
| CSS0045924 | 3.2222 | 2.4639 | 2.6226 | 37.3025 | 37.678 | 42.9562 | 0.2388 | 0.2727 | 0.3219 | 3.5974 | 4.8184 | 3.6276 |
| CSS0048905 | 138.6004 | 137.9064 | 136.3253 | 25.7837 | 25.9677 | 31.5039 | 12.8804 | 15.0964 | 15.411 | 1.0742 | 0.6289 | 1.2898 |
| CSS0033554 | 3.0209 | 3.3656 | 3.4335 | 0.6821 | 0.9141 | 0.2089 | 1.7123 | 2.8017 | 2.6865 | 0.6242 | 0.8633 | 0.9571 |
| CSS0014132 | 4.7249 | 4.7924 | 5.295 | 32.2061 | 27.1077 | 30.4391 | 1.7813 | 1.8373 | 0.8002 | 3.6723 | 3.2619 | 2.7258 |
| CSS0022212 | 4.4112 | 7.0608 | 16.4651 | 701.0985 | 712.2577 | 830.9234 | 0.194 | 0.1289 | 0.0634 | 6.3467 | 5.0971 | 6.2382 |
| CSS0010687 | 22.1163 | 21.0779 | 28.0886 | 155.4733 | 160.0358 | 143.9144 | 20.6391 | 22.5539 | 17.0169 | 143.7856 | 134.2345 | 122.7106 |
| CSS0041663 | 13.9479 | 14.0976 | 14.949 | 263.0996 | 259.373 | 261.8029 | 17.6407 | 13.8432 | 14.8332 | 68.0365 | 66.635 | 63.6188 |
| CSS0028235 | 4.7374 | 12.2308 | 11.4586 | 346.1077 | 339.7703 | 358.9826 | 1.9305 | 3.7419 | 1.4063 | 22.2595 | 21.1012 | 17.5431 |
| CSS0034690 | 82.3039 | 90.9007 | 107.7993 | 30.5575 | 30.7608 | 31.1267 | 10.9447 | 17.2279 | 17.6121 | 0.7948 | 0.6268 | 1.0263 |
| CSS0009596 | 92.8543 | 109.7027 | 109.3805 | 2.4611 | 3.0589 | 3.3561 | 156.1282 | 225.4368 | 290.5149 | 42.6146 | 39.1225 | 35.7671 |
| CSS0011093 | 29.7511 | 26.5633 | 32.5423 | 4.5832 | 4.8526 | 4.8804 | 297.8467 | 333.9865 | 465.3186 | 79.1466 | 74.7943 | 103.375 |
| CSS0039811 | 35.1823 | 37.2049 | 35.3206 | 8.4871 | 9.2463 | 8.5721 | 60.5257 | 48.2941 | 68.4266 | 13.6643 | 14.9623 | 14.226 |
| CSS0035437 | 16.9662 | 22.8411 | 20.8243 | 1.167 | 4.4309 | 1.1796 | 39.8337 | 38.488 | 31.4277 | 1.9579 | 1.518 | 2.238 |
| CSS0041635 | 10.1487 | 9.531 | 13.0691 | 300.3151 | 310.689 | 307.7104 | 0.5545 | 0.4266 | 0.4457 | 2.3284 | 2.3276 | 1.7001 |
| CSS0043088 | 11.2005 | 12.734 | 10.8387 | 550.6116 | 548.6677 | 595.0951 | 15.8879 | 16.4105 | 8.2198 | 56.3915 | 54.4391 | 46.1116 |

Table S4 Characteristics of Illumina second-generation sequencing data of FCC

| Library | Data (Gb) | Depth (×) | GC (%) | Q20 (%) | Q30 (%) |
| --- | --- | --- | --- | --- | --- |
| FCC | 221.01 | 73.83 | 37.53 | 97.9 | 94.54 |
| Total | 221.01 | 73.83 | 37.53 | --- | --- |

Table S5 Information of KASP

| KASP | FAM | VIC | COM | Chr_position | Mutation |
| --- | --- | --- | --- | --- | --- |
| CsSNP36770958 | CATTAAACAAGGGGAAGATTTACATA | CATTAAACAAGGGGAAGATTTACATG | GCTTCTCTTACAACAGGAGGGCTTGTC | Chr1_36770958 | [A/G] |
| CsSNP36771094 | GTGTTGATACTTCGTGGTAGGCTAT | GTGTTGATACTTCGTGGTAGGCTAG | GCTCCCCATGTCTGAAGGATCAGAAG | Chr1_36771094 | [T/G] |
| CsSNP36772011 | GATTGATCGATGAAGATAAATTAGACA | GATTGATCGATGAAGATAAATTAGACG | CTTTCACTTCCTCAAGTGATCC | Chr1_36772011 | [A/G] |
| CsSNP36772215 | CATATAGTTTGTCCATGATTTCATC | CATATAGTTTGTCCATGATTTCATA | CACAGAGCCTATAATTTCAAACCAG | Chr1_36772215 | [C/A] |

Table S6 Genotype and alkaloid contents of 80 tea accessions

| Number | Species | Origin | Genotype | Caffeine (%) | Theobromine (%) |
| --- | --- | --- | --- | --- | --- |
| FCC_1 | *Camellia fangchengensis* | Guangxi | AG | 0.670 | 4.777 |
| FCC_2 | *Camellia fangchengensis* | Guangxi | AG | 0.746 | 4.521 |
| FCC_3 | *Camellia fangchengensis* | Guangxi | AG | 0.670 | 4.109 |
| FCC_4 | *Camellia fangchengensis* | Guangxi | AG | 0.864 | 3.983 |
| FCC_5 | *Camellia fangchengensis* | Guangxi | AG | 0.860 | 4.531 |
| FCC_6 | *Camellia fangchengensis* | Guangxi | AG | 0.720 | 3.890 |
| FCC_7 | *Camellia fangchengensis* | Guangxi | AG | 0.714 | 4.467 |
| FCC_8 | *Camellia fangchengensis* | Guangxi | AG | 0.747 | 4.415 |
| FCC_9 | *Camellia fangchengensis* | Guangxi | AG | 0.587 | 3.267 |
| FCC_10 | *Camellia fangchengensis* | Guangxi | AG | 0.906 | 4.208 |
| FCC_11 | *Camellia fangchengensis* | Guangxi | AG | 0.783 | 3.869 |
| FCC_12 | *Camellia fangchengensis* | Guangxi | AG | 0.711 | 3.585 |
| FCC_13 | *Camellia fangchengensis* | Guangxi | AG | 0.742 | 4.768 |
| FCC_14 | *Camellia fangchengensis* | Guangxi | AG | 0.710 | 4.725 |
| FCC_15 | *Camellia fangchengensis* | Guangxi | AG | 0.666 | 4.120 |
| FCC_16 | *Camellia fangchengensis* | Guangxi | AG | 0.769 | 4.601 |
| FCC_17 | *Camellia fangchengensis* | Guangxi | AG | 0.740 | 4.094 |
| FCC_18 | *Camellia fangchengensis* | Guangxi | AG | 0.608 | 3.314 |
| FCC_19 | *Camellia fangchengensis* | Guangxi | AG | 0.756 | 4.786 |
| FCC_20 | *Camellia fangchengensis* | Guangxi | AG | 0.690 | 4.796 |
| FCC_21 | *Camellia fangchengensis* | Guangxi | AG | 0.625 | 3.824 |
| FCC_22 | *Camellia fangchengensis* | Guangxi | AG | 0.649 | 4.454 |
| FCC_23 | *Camellia fangchengensis* | Guangxi | AG | 0.702 | 4.316 |
| FCC_24 | *Camellia fangchengensis* | Guangxi | AG | 0.618 | 4.062 |
| FCC_25 | *Camellia fangchengensis* | Guangxi | AG | 0.902 | 4.438 |
| FCC_26 | *Camellia fangchengensis* | Guangxi | AG | 0.814 | 4.355 |
| FCC_27 | *Camellia fangchengensis* | Guangxi | AG | 0.621 | 3.956 |
| FCC_28 | *Camellia fangchengensis* | Guangxi | AG | 0.753 | 4.706 |
| FCC_29 | *Camellia fangchengensis* | Guangxi | AG | 0.735 | 5.135 |
| FCC_30 | *Camellia fangchengensis* | Guangxi | AG | 0.573 | 4.019 |
| FCC_31 | *Camellia fangchengensis* | Guangxi | AG | 0.794 | 4.834 |
| FCC_32 | *Camellia fangchengensis* | Guangxi | AG | 0.746 | 4.883 |
| FCC_33 | *Camellia fangchengensis* | Guangxi | AG | 0.669 | 4.430 |
| FCC_34 | *Camellia fangchengensis* | Guangxi | AG | 0.731 | 4.042 |
| FCC_35 | *Camellia fangchengensis* | Guangxi | AG | 0.670 | 3.415 |
| FCC_36 | *Camellia fangchengensis* | Guangxi | AG | 0.778 | 4.413 |
| FCC_37 | *Camellia fangchengensis* | Guangxi | AG | 0.810 | 4.536 |
| FCC_38 | *Camellia fangchengensis* | Guangxi | AG | 0.741 | 4.847 |
| FCC_39 | *Camellia fangchengensis* | Guangxi | AG | 0.662 | 3.645 |
| FCC_40 | *Camellia fangchengensis* | Guangxi | AG | 0.573 | 4.019 |
| F1_pop_1 | *Camellia sinensis* | Hangzhou | AA | 2.923 | 0.080 |
| F1_pop_2 | *Camellia sinensis* | Hangzhou | AA | 3.041 | 0.090 |
| F1_pop_3 | *Camellia sinensis* | Hangzhou | AA | 3.758 | 0.099 |
| F1_pop_4 | *Camellia sinensis* | Hangzhou | AA | 3.766 | 0.107 |
| F1_pop_5 | *Camellia sinensis* | Hangzhou | AA | 3.566 | 0.111 |
| F1_pop_6 | *Camellia sinensis* | Hangzhou | AA | 3.264 | 0.112 |
| F1_pop_7 | *Camellia sinensis* | Hangzhou | AA | 3.696 | 0.119 |
| F1_pop_8 | *Camellia sinensis* | Hangzhou | AA | 3.066 | 0.128 |
| F1_pop_9 | *Camellia sinensis* | Hangzhou | AA | 3.465 | 0.129 |
| F1_pop_10 | *Camellia sinensis* | Hangzhou | AA | 3.416 | 0.133 |
| F1_pop_11 | *Camellia sinensis* | Hangzhou | AA | 3.271 | 0.137 |
| F1_pop_12 | *Camellia sinensis* | Hangzhou | AA | 3.579 | 0.138 |
| F1_pop_13 | *Camellia sinensis* | Hangzhou | AA | 3.278 | 0.139 |
| F1_pop_14 | *Camellia sinensis* | Hangzhou | AA | 4.111 | 0.158 |
| F1_pop_15 | *Camellia sinensis* | Hangzhou | AA | 3.151 | 0.159 |
| F1_pop_16 | *Camellia sinensis* | Hangzhou | AA | 3.326 | 0.175 |
| F1_pop_17 | *Camellia sinensis* | Hangzhou | AA | 3.841 | 0.175 |
| F1_pop_18 | *Camellia sinensis* | Hangzhou | AA | 3.483 | 0.177 |
| F1_pop_19 | *Camellia sinensis* | Hangzhou | AA | 2.771 | 0.187 |
| F1_pop_20 | *Camellia sinensis* | Hangzhou | AA | 3.355 | 0.188 |
| F1_pop_21 | *Camellia sinensis* | Hangzhou | AA | 3.462 | 0.207 |
| F1_pop_22 | *Camellia sinensis* | Hangzhou | AA | 3.378 | 0.212 |
| F1_pop_23 | *Camellia sinensis* | Hangzhou | AA | 3.538 | 0.212 |
| F1_pop_24 | *Camellia sinensis* | Hangzhou | AA | 3.287 | 0.219 |
| F1_pop_25 | *Camellia sinensis* | Hangzhou | AA | 3.451 | 0.220 |
| F1_pop_26 | *Camellia sinensis* | Hangzhou | AA | 3.426 | 0.220 |
| F1_pop_27 | *Camellia sinensis* | Hangzhou | AA | 3.484 | 0.227 |
| F1_pop_28 | *Camellia sinensis* | Hangzhou | AA | 3.874 | 0.228 |
| F1_pop_29 | *Camellia sinensis* | Hangzhou | AA | 2.997 | 0.230 |
| F1_pop_30 | *Camellia sinensis* | Hangzhou | AA | 3.668 | 0.233 |
| F1_pop_31 | *Camellia sinensis* | Hangzhou | AA | 3.474 | 0.237 |
| F1_pop_32 | *Camellia sinensis* | Hangzhou | AA | 3.654 | 0.243 |
| F1_pop_33 | *Camellia sinensis* | Hangzhou | AA | 2.966 | 0.246 |
| F1_pop_34 | *Camellia sinensis* | Hangzhou | AA | 3.366 | 0.247 |
| F1_pop_35 | *Camellia sinensis* | Hangzhou | AA | 3.117 | 0.255 |
| F1_pop_36 | *Camellia sinensis* | Hangzhou | AA | 4.185 | 0.260 |
| F1_pop_37 | *Camellia sinensis* | Hangzhou | AA | 3.563 | 0.262 |
| F1_pop_38 | *Camellia sinensis* | Hangzhou | AA | 3.225 | 0.270 |
| F1_pop_39 | *Camellia sinensis* | Hangzhou | AA | 3.229 | 0.298 |
| F1_pop_40 | *Camellia sinensis* | Hangzhou | AA | 2.916 | 0.298 |

Table S7 Sensory evaluation of different tea types processed from new shoots of FCC

| **Tea type** | **Appearance** | **Liquor color** | **Aroma** | **Taste** | **Infused leaves** |
| --- | --- | --- | --- | --- | --- |
| GT | Heavy and tight;  slightly twist;  Brown on white side; full of silver sharp ends | Pale yellowwish green; Reddish | Sweet scent;  Slightly pekoe flavour | Pure elegant;  Slightly astringent | Thick and soft;  Red stem |
| WT | Strong and head;  full of gray green sharp ends | Bright yellow; clear and bright | Sweet fruit fragrance;  Dried plum smell;  Slightly pekoe flavour | Sweet;  Slightly sour;  Engender liquid | Thick;  Grayish brown |
| BT | Loose;  tea with fine hairs;  black and gold bud body | Orange-red;  Slightly cloudy | Sweet-smelling;  green miscellaneous smell; Stuffy | Relatively sweet; slightly dull | Soft;  Slightly uniform;  Redder |

**Supplementary Figure**

| 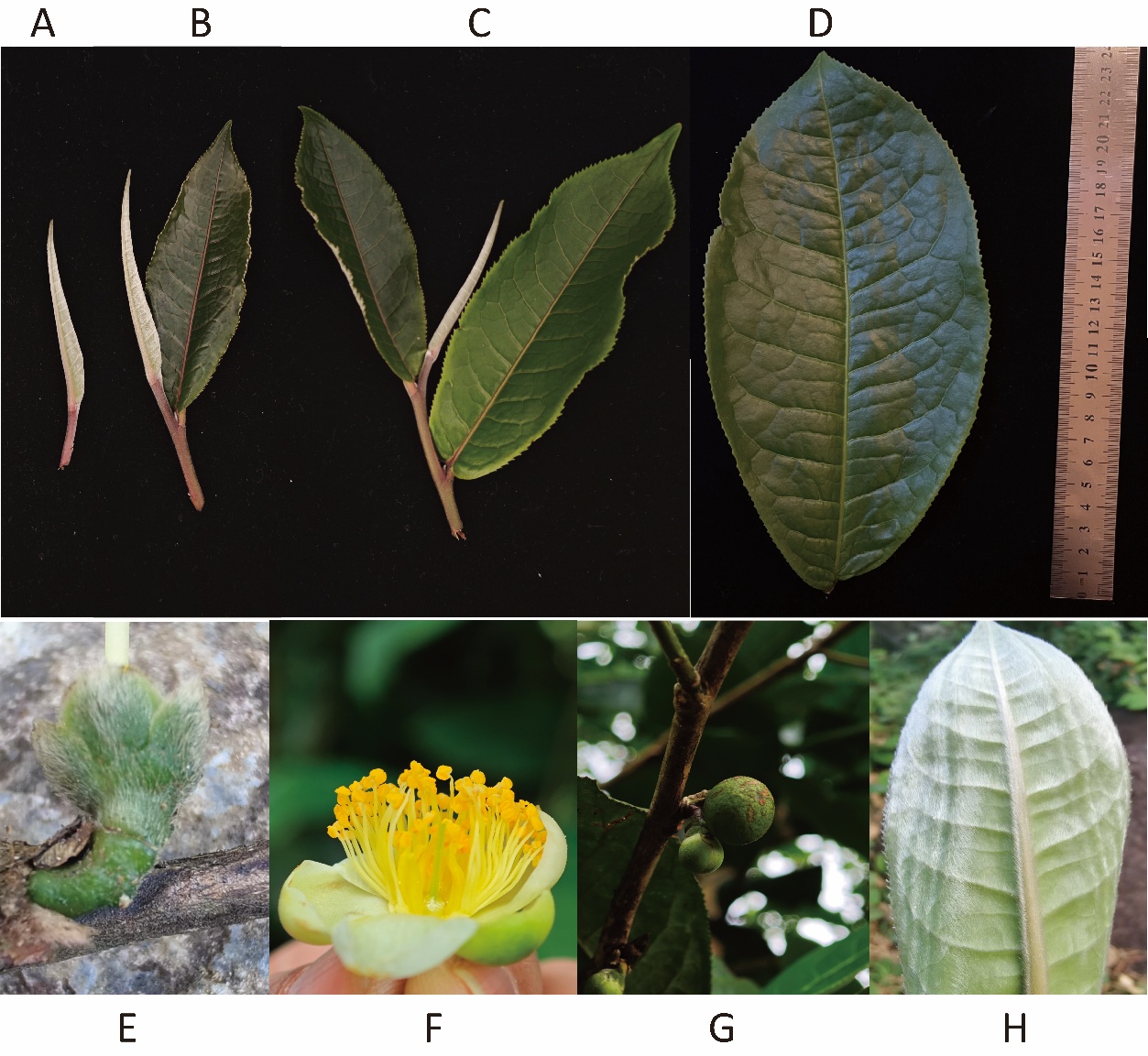 |
| --- |
| **Figure S1.** Analysis of morphological characteristics of *C. fangchengensis* (FCC)  A. Bud; B. one bud and one leaf; C. one bud and two leaves; D. mature leaf; E. calyx; F. flower; G. fruit; H. trichomes of on the back of the leaf. |

| 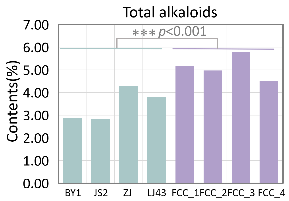A | 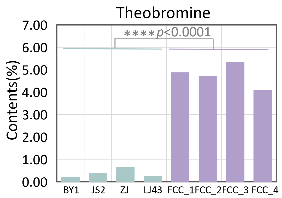B | 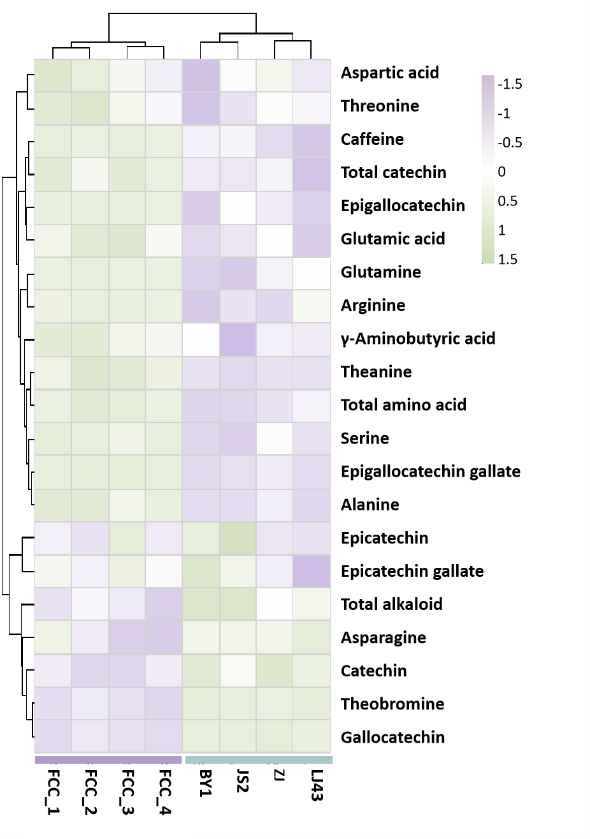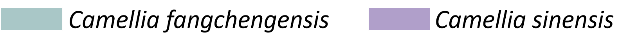I |
| --- | --- | --- |
| 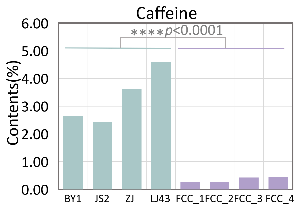C | 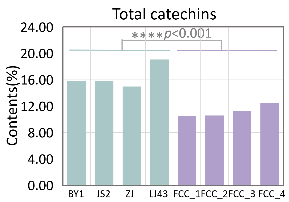D |  |
| 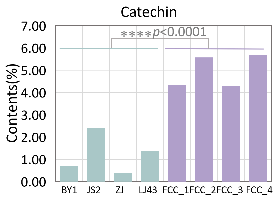E | 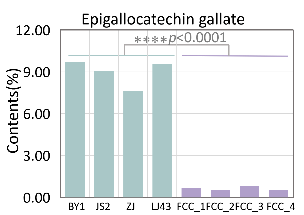F |  |
| 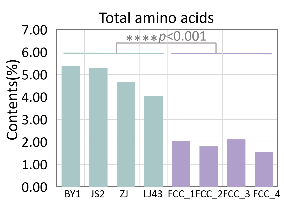G | 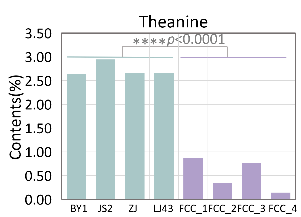H |  |
| **Figure S2. Biochemical component analysis of *C.*** ***fangchengensis* and *C. sinensis*.**  A. total alkaloid; B. theobromine; C. caffeine; D. total catechin; E. catechin; F. epigallocatechin gallate; G. total amino acid; H. theanine; I. heatmap of all biochemical components. | | |

| **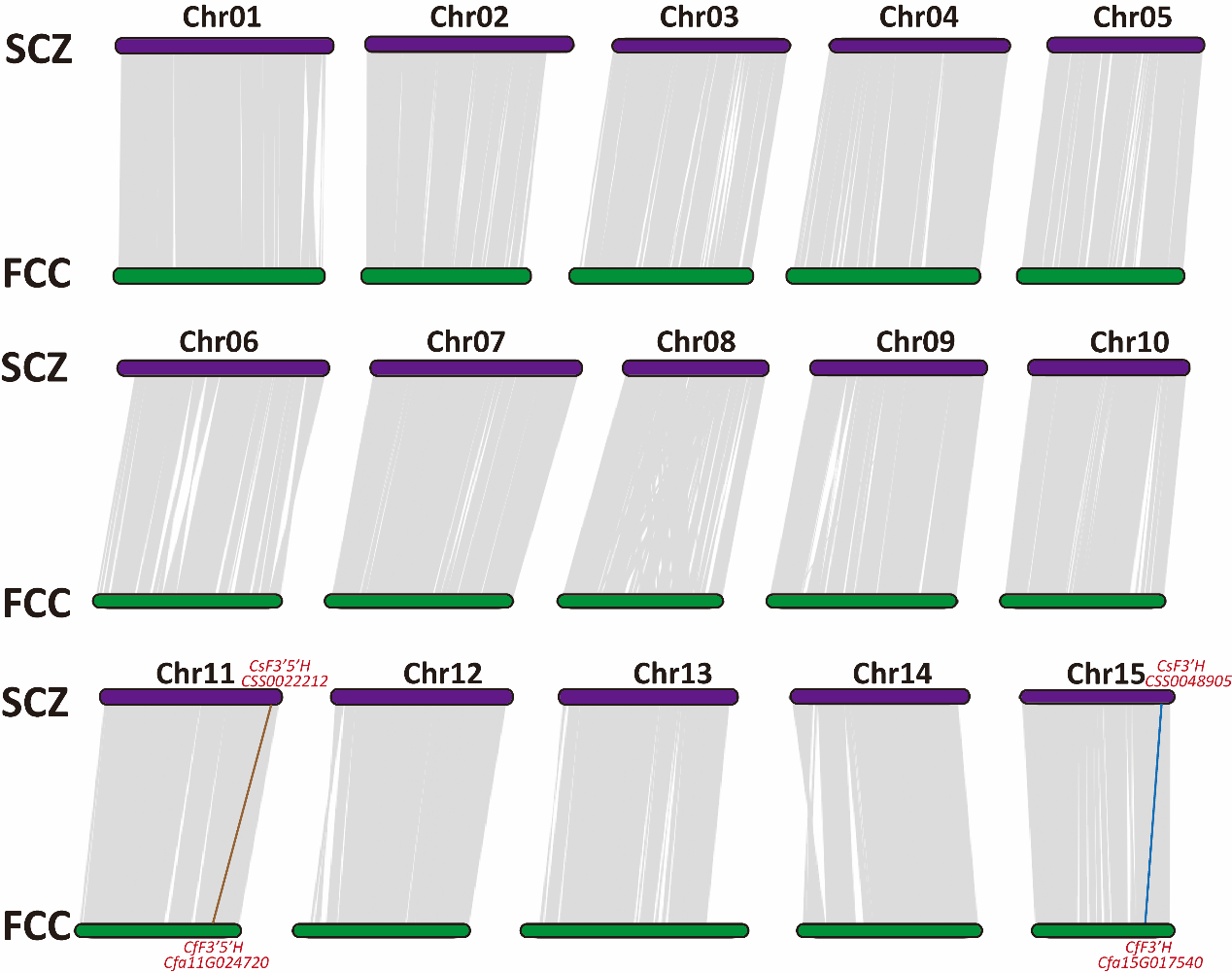** |
| --- |
| **Figure S3. Collinearity analysis between genomes of *C.*** ***fangchengensis* (FCC) and *C. sinensis* cv. *Shuchazao* (SCZ).** |

| 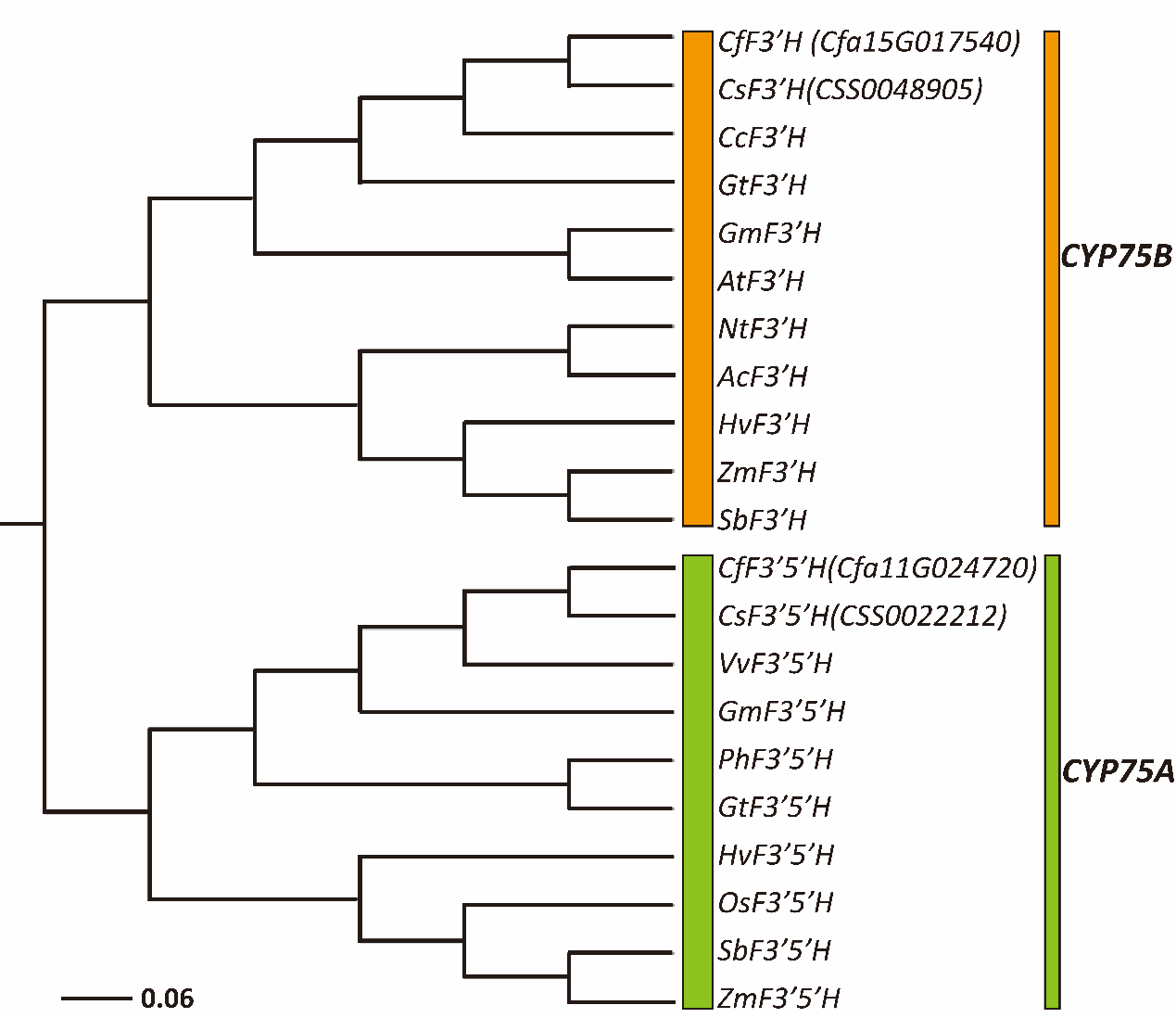 |
| --- |
| **Figure S4. Phylogenetic tree of *F3’5’H* and *F3’H* from tea plant and other plant species.**  **Note:** *Gm: Glycine max; Vv: Vitis vinifera; Gt:* *Gentiana triflora; Ph:Petunia hybrida; Hv:Hordeum vulgare; Os:* *Oryza sativa; Zm: Zea mays; Sb: Sorghum bicolor; Nt: Narcissus tazetta ; Ac: Allium cepa; At: Arabidopsis thaliana; Cc: Callistephus chinensis* |

| 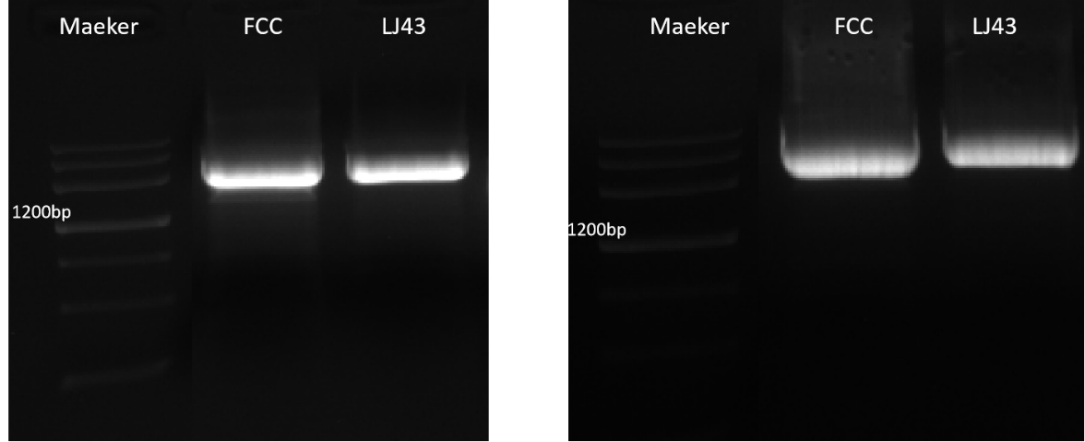A B |
| --- |
| 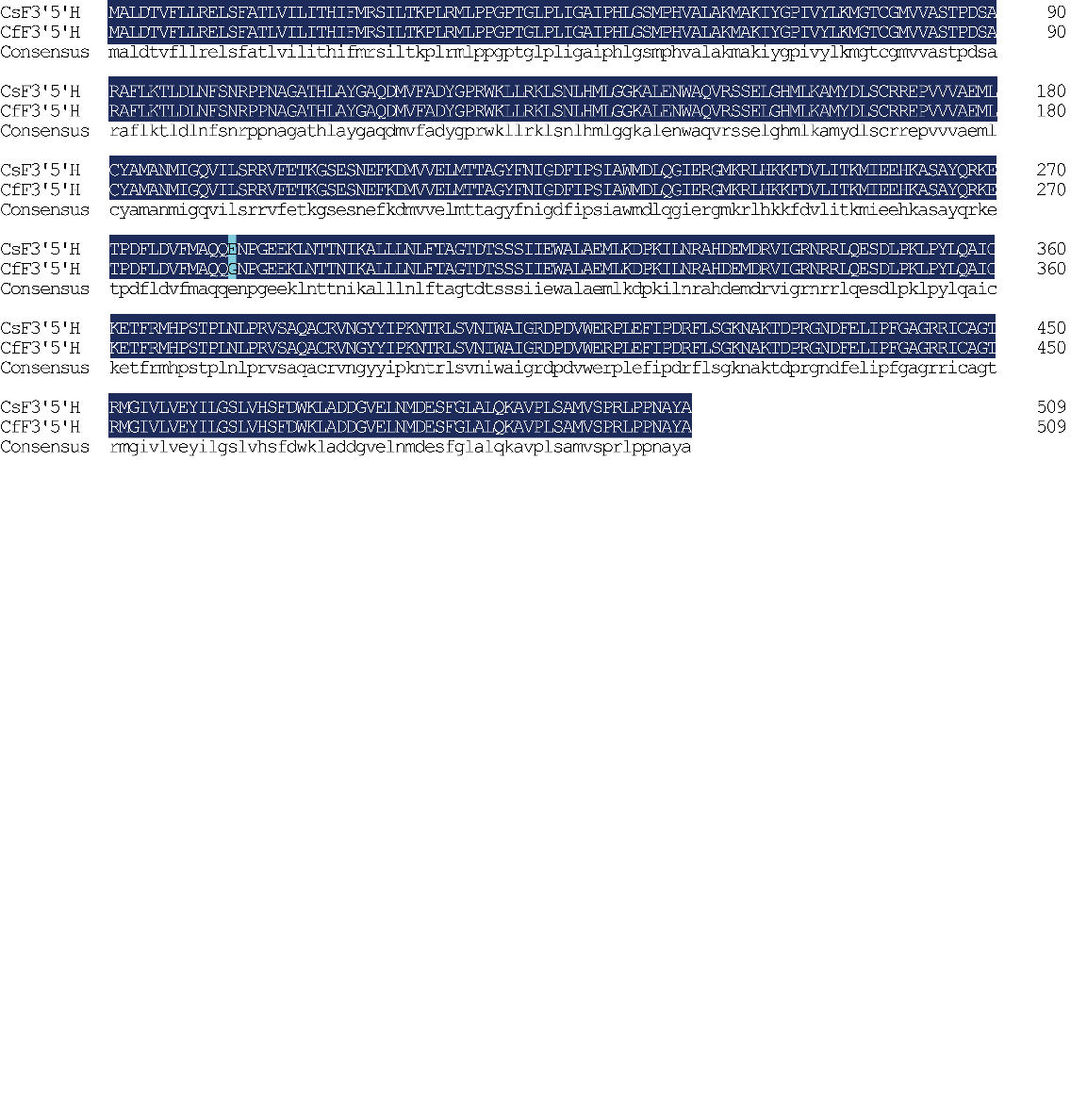C |
| 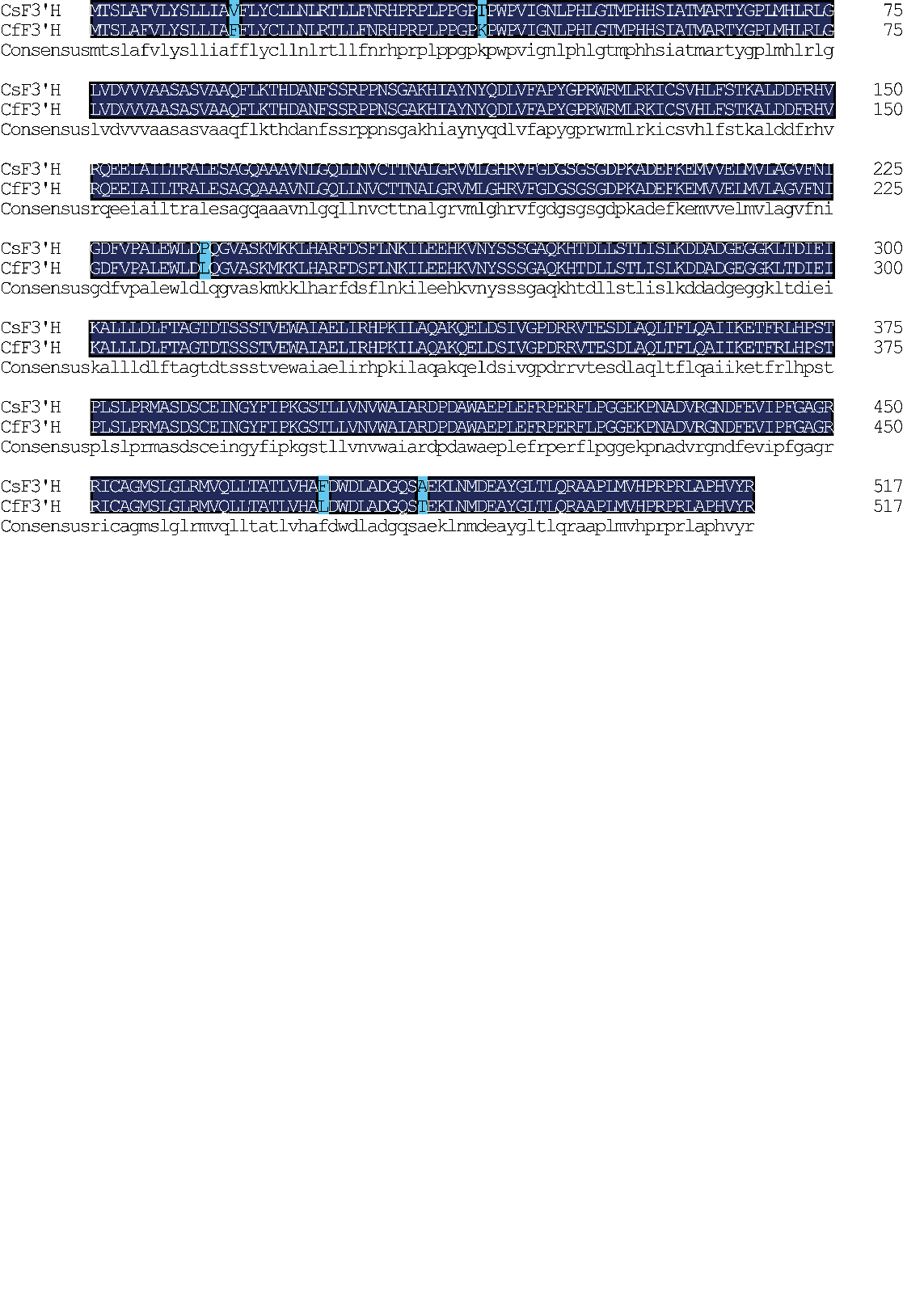D |
| **Figure S5. Sequence analysis of *F3'5H* and *F3'H* genes in *C.*** ***fangchengensis* (FCC) and *C. sinensis* cv. *Longjing 43* (LJ43).**  A. Cloning of *F3'5H* gene; B. Cloning of *F3'5H* gene; C. Comparative analysis of cloned *F3'5'H* gene sequences; D. Comparative analysis of cloned *F3'H* gene sequences. |

| 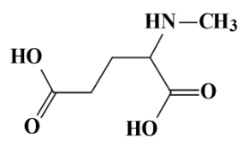 | 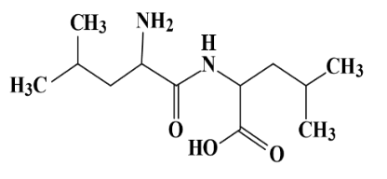 | 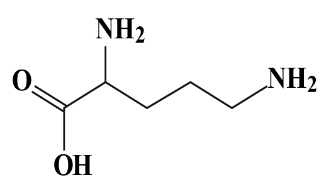 |
| --- | --- | --- |
| **N-Methyl-L-Glutamate** | **L-Leucyl-L-Leucine** | **L-Ornithine** |
| 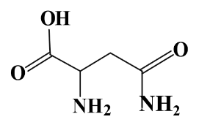 | 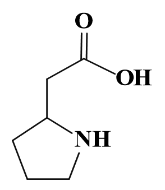 |  |
| **L-Asparagine** | **Homoproline** |  |
| 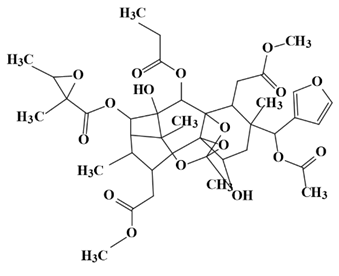 | 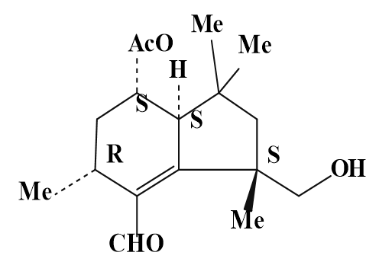 | 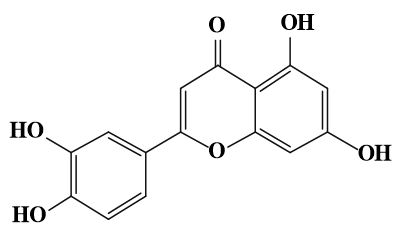 |
| **Swietenialide D** | **Botryenalol** | **Luteolin 7-rutinoside-4'-O-Rhamnoside** |
| 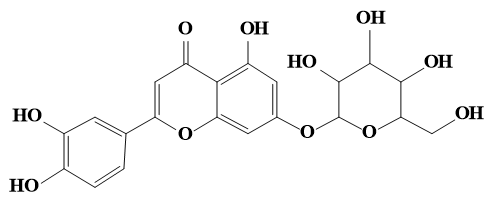 | 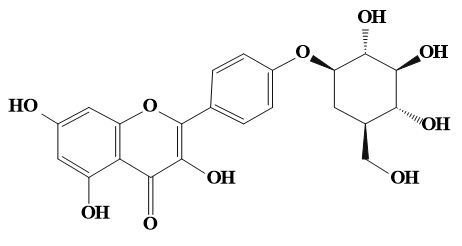 | 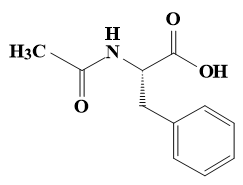 |
| **Luteolin-7-O-glucoside** | **Kaempferol-4'-O-glucoside** | **N-Acetyl-DL-phenylalanine** |
| 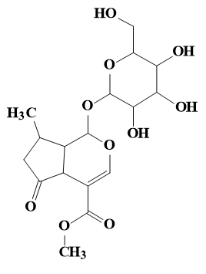 | 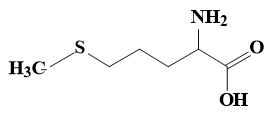 | 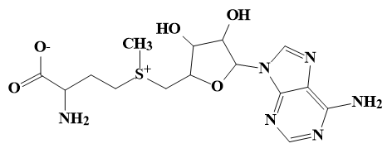 |
| **Verbenalin** | **L-Homomethionine** | **S-Adenosylmethionine** |
| 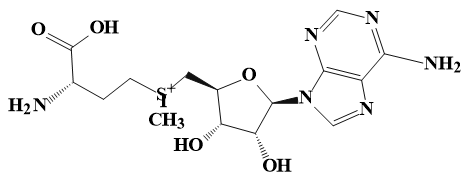 | 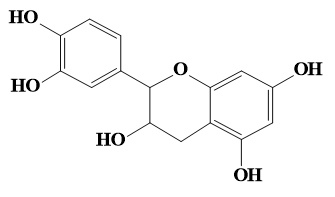 | 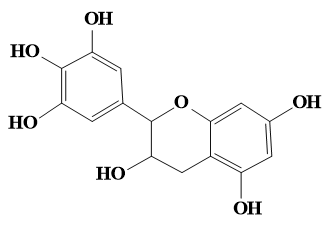 |
| **S-(5'-Adenosyl)-L-methionine** | **Epicatechin** | **Epigallocatechin** |
| 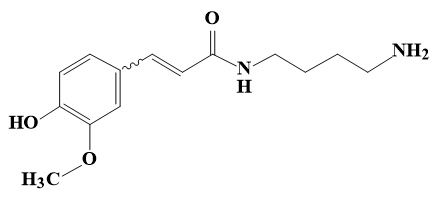 | 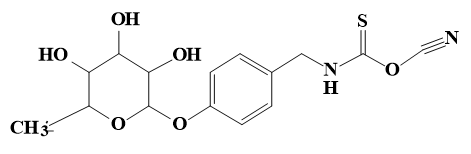 |  |
| **N-Feruloylputrescine** | **niazidin** |  |
| **Figure S6. Structural formulas of characteristic metabolites from different tea samples types produced from *C. fangchengensis* new shoots.** | | |
